# Uncovering miRNA-mRNA Regulatory Modules in Developing Xylem of *Pinus massoniana* via Small RNA and Degradome Sequencing

**DOI:** 10.3390/ijms221810154

**Published:** 2021-09-21

**Authors:** Tengfei Shen, Mengxuan Xu, Haoran Qi, Yuanheng Feng, Zhangqi Yang, Meng Xu

**Affiliations:** 1 Co-Innovation Center for Sustainable Forestry in Southern China, Key Laboratory of Forest Genetics and Biotechnology Ministry of Education, Nanjing Forestry University, Nanjing 210037, China; stf.njfu@outlook.com (T.S.); atrecee@outlook.com (M.X.); haoranqi0430@gmail.com (H.Q.); 2 Guangxi Key Laboratory of Superior Timber Trees Resource Cultivation, Guangxi Forestry Research Institute, Nanning 530002, China; nanyuan05@163.com (Y.F.); yangzhangqi@163.com (Z.Y.)

**Keywords:** masson pine, developing xylem, protoplasts, cell wall, small RNA, degradome

## Abstract

Xylem is required for the growth and development of higher plants to provide water and mineral elements. The thickening of the xylem secondary cell wall (SCW) not only improves plant survival, but also provides raw materials for industrial production. Numerous studies have found that transcription factors and non-coding RNAs regulate the process of SCW thickening. *Pinus massoniana* is an important woody tree species in China and is widely used to produce materials for construction, furniture, and packaging. However, the target genes of microRNAs (miRNAs) in the developing xylem of *P. massoniana* are not known. In this study, a total of 25 conserved miRNAs and 173 novel miRNAs were identified via small RNA sequencing, and 58 differentially expressed miRNAs were identified between the developing xylem (PM_X) and protoplasts isolated from the developing xylem (PM_XP); 26 of these miRNAs were significantly up-regulated in PM_XP compared with PM_X, and 32 were significantly down-regulated. A total of 153 target genes of 20 conserved miRNAs and 712 target genes of 113 novel miRNAs were verified by degradome sequencing. There may be conserved miRNA-mRNA modules (miRNA-*MYB*, miRNA-*ARF*, and miRNA-*LAC*) involved in softwood and hardwood formation. The results of qRT-PCR-based parallel validation were in relatively high agreement. This study explored the potential regulatory network of miRNAs in the developing xylem of *P. massoniana* and provides new insights into wood formation in coniferous species.

## 1. Introduction

The vascular tissue of higher plants consists mainly of the phloem and xylem. The xylem transports water and inorganic salts throughout plants and provides mechanical support for plant growth, and it is the basic channel for material and energy transport for plant organs [1]. The secondary xylem arises from the vascular layer, and it is generally present when vascular plants undergo secondary growth. The secondary xylem, which is also known as wood, is the raw material for paper, construction and textile industries, and it is closely related to human productivity and life. The secondary xylem includes vessels, parenchyma cells, and fibers. Vessels and fiber cells undergo secondary cell wall (SCW) thickening. This process increases the mechanical strength of plants and provides abundant lignocellulosic biomass material for human activities and materials [2,3]. The SCW of plants comprises most of the photosynthesis products that accumulate in plants, and it is the main raw material of wood.

The main components of wood include cellulose, hemicellulose, and lignin. Cellulose accounts for almost 1/3 of the dry weight of the plant, and it is the most important component of SCWs. Cellulose is also the most abundant and renewable natural organic polymer in nature [4]. Cellulose is a linear polyglucan formed by D-glucose monomers linked by β-1,4-glycosidic bonds, and its synthesis machinery is controlled by the cellulose synthase (CESA) complex, which is located in the plasma membrane [5]. The cellulose synthase complex (CSC) is composed of at least 18 CESA subunits [5]. Hemicellulose is the second major carbohydrate that forms the structure of SCW, and it is a branched heteropolysaccharide consisting of two or more monosaccharides linked in different ways [6]. Different types of plants have different hemicellulose structures. The hemicellulose monosaccharides in broadleaf trees, herbaceous plants, and plants from which herbal medicines are obtained are mainly xylose monosaccharides, and the hemicellulose monosaccharides in coniferous trees primarily include arabinose, galactose, and mannose [7]. Lignin is synthesized during SCW formation in plants. Lignin provides mechanical support and allows the transport of water and nutrients over long distances, which enables trees to grow to great heights and prevents pathogen invasion [8,9]. The biosynthesis of lignin involves a very complex process. Research showed that the shikimic acid pathway, phenylpropionic acid pathway, and other specific pathways were necessary for lignin biosynthesis [9].

Forest trees provide most of the wood needed for human production. Thickened SCWs constitute a major component of wood, and a three-level regulatory network of transcription factors (TFs) tightly control their formation. The investigation of the process of wood synthesis, specifically SCW thickening, will improve our understanding of the mechanisms governing plant cell development and wood formation. The regulatory network of wood formation, mainly the NAC-MYB TF network, was elucidated recently. TFs in this regulatory network containing a NAC structural domain act upstream and are switches of the SCW synthesis regulatory network. AtMYB46 and AtMYB83 act in the second layer of the SCW biosynthesis regulatory network and are two redundant R2R3-MYB TFs that are the direct target genes of NAC and key genes for SCW formation in *Arabidopsis thaliana*. A series of *MYB* genes and other types of TFs play roles at the third level [10,11,12,13,14,15,16,17]. Non-coding RNAs, represented by microRNAs (miRNAs), also regulate SCW thickening.

Plant small RNAs (sRNAs) are divided into two categories based on their precursor structures: microRNAs (miRNAs) and small interfering RNAs (siRNAs) [18]. Plant miRNAs are composed of a class of sRNAs with a length of 20~24 nt, and these molecules target mRNAs via a seed region at positions 2~7 at the 5′ end [19]. Shorter miRNAs cleave target mRNAs or inhibit the translation of their target mRNAs into proteins, and longer miRNAs of 24 nt are involved in DNA methylation [20]. The discovery that miRNAs target enhancers to activate gene expression debunked the notion that miRNAs only inhibited gene expression [21,22]. MiRNAs are involved in the production of other sRNAs, and the *TAS1/2/3/4* genes of *A. thaliana* were studied in depth. *TAS1/2*, *TAS3*, and *TAS4* are triggered by miR173, miR390, and miR828, respectively, and the resulting target genes of phased secondary small interfering RNAs (phasiRNAs) are *pentatricopeptide-repeat* (*PPR*), *auxin response factors* (*ARF*), and *MYB* [23]. PhasiRNAs are targeted to the original transcript in *cis* or to other transcripts in *trans* [24]. Primary miRNAs (pri-miRNAs) encode functional peptides, and three small open reading frames (sORFs) were found within pri-miR171 in *Vitis vinifera*, one of which encodes a small peptide that enhances the expression of the *vvi-miR171* gene [25,26].

*P. massoniana* belongs to the *Pinus* genus, and it is widely distributed in southern China. This plant adapts to relatively poor soil conditions. Its wood is rich in cellulose, which may be used to make paper pulp, and it has high economic value [27]. Previous studies showed that a variety of miRNAs exist in angiosperms and these miRNAs were studied extensively. However, few studies on miRNAs were reported for gymnosperms. Since scientists discovered the first miRNA in *Caenorhabditis elegans* in 1993, a database for storing the sequences of mature miRNAs and precursors of miRNAs, miRBase, is home to sequences of more than 38,000 miRNAs from 271 species, but no sequences of miRNAs of *P. massoniana* are included [28,29]. Therefore, the possible roles of miRNAs in *P. massoniana* are not clear. This study combined sRNA and degradome sequencing to explore the potential regulatory network of miRNAs involved in xylem development in *P. massoniana* and provide new insights into wood formation in coniferous species.

## 2. Results

### 2.1. sRNAs Generated from Sequencing

**Figure 1:**
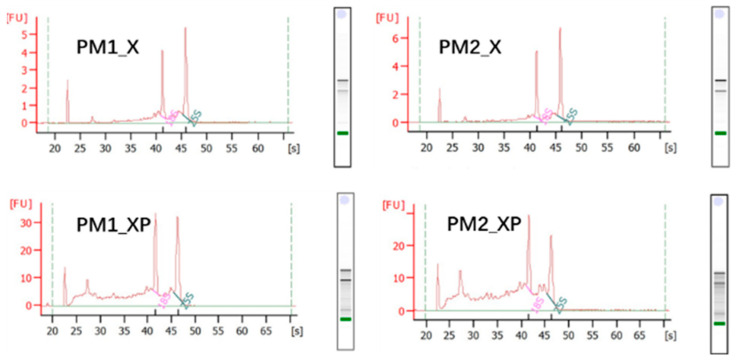
RNA quality of the developing xylem (PM_X) and protoplasts isolated from developing xylem (PM_XP).

In this study, high quality RNA was extracted from developing xylem (PM_X) and protoplasts isolated from developing xylem (PM_XP) for sRNA sequencing (Figure 1). We obtained 75,185,529 raw reads and 72,664,728 clean reads through sRNA sequencing, with mean Q20 and Q30 values of 97.68% and 94.77%, respectively (Table 1). Previous studies reported that plant sRNAs were 18~30 nt in length, most of which were 21~22 nt [30]. Our analysis revealed that the lengths of the sRNAs of the four libraries were similarly distributed, and their lengths ranged from 18~30 nt. Most of the sRNAs were 21 nt in length, and these sRNAs constituted 51.83, 43.61, 19.46, and 22.67% of the distributions within the four libraries (Figure S1). More than 13 million reads were compared to the *P. massoniana* transcriptome, and 155,015 reads per library were identified on average as conserved miRNAs, while 164,185 reads were novel miRNAs (Table 1).

### 2.2. Identification of miRNAs Involved in Developing Xylem

**Figure 2:**
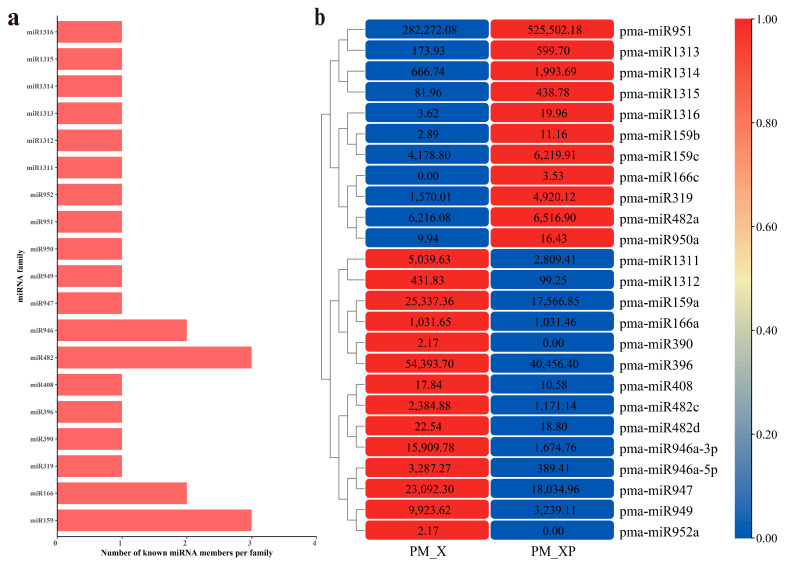
The conserved miRNAs in *P. massoniana*. (**a**) Member numbers of conserved miRNAs. (**b**) The expression of conserved miRNAs. MiRNA expression was log_10_ (TPM + 1) processed and normalized, and the detailed expression of miRNAs is shown in black font in the figure.

Detailed information about the mature and precursor sequences of 198 miRNAs is shown in Table S1 and Additional file S1. Only 25 conserved miRNAs were found in PM_XP and PM_X, and these miRNAs belonged to 19 families (Figure 2a, Table S1). We used the transcripts per million (TPM) method to measure the abundance of miRNAs. The abundances of the 25 conserved miRNAs varied greatly. The TPM values of pma-miR159a, pma-miR396, pma-miR947, and pma-miR951 were greater than 10,000, and the values of pma-miR1316, pma-miR159b, pma-miR390, pma-miR950a, and pma-miR952a were lower than 10. The TPM difference between different members of the same miRNA family, such as the pma-miR159 family, was also very large. The TPM value of pma-miR159a in PM_XP and PM_X was greater than 10,000, but the TPM value of pma-miR159b was extremely low (Figure 2b, Table S2).

**Figure 3:**
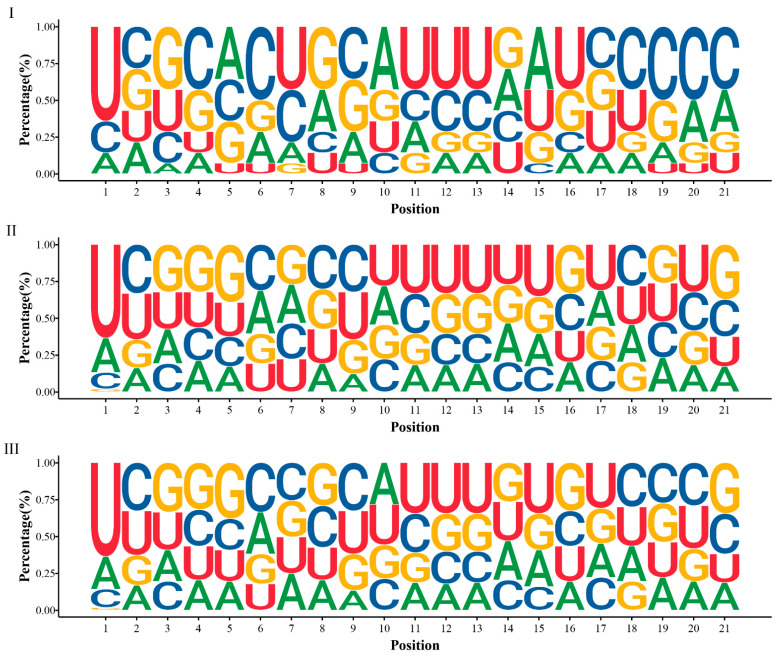
Base ratio of 21 nt miRNAs at each position, (**I**) for conserved miRNAs, (**II)** for novel miRNAs, and (**III)** for all miRNAs. For each position, larger percentages of bases are closer to 1 on the y-axis.

We also identified 173 novel miRNAs that ranged from 18~24 nt in length (Table S1), which is consistent with the criteria established in previous studies [30]. Most of the novel miRNAs were 21 nt in length, and these miRNAs accounted for greater than 50% of the total amount of novel miRNAs (Figure S2). MiRNAs have relatively strong base preference and understanding the base preference of miRNAs can lead to an improved understanding of the characteristics of *P. massoniana* miRNAs. We found that the most common base at the 5′ end of the miRNA of *P. massoniana* was uridine (U) (Figure 3). The cleavage site of the target gene is generally between the 10th and 11th nucleotides composing the miRNA [18]. The dominant nucleotides at positions 10 and 11 of the conserved miRNAs were adenine (A) and thymine (U), respectively, and most of the nucleotides of the novel miRNAs were thymine (U) at positions 10 and 11 (Figure 3). T-tests found a significant difference in the base distribution between conserved and novel miRNAs at positions 10 and 11 (p adj = 0.000044 for position 10, and p adj = 0.0058 for position 11). In contrast, for all of the miRNAs present in the developing xylem of *P. massoniana*, we found a strong preference for U at position 1, A at position 10, and U at position 11 of the miRNA, which is consistent with previous studies. Most plant miRNAs start with U at the 5′ end, which is associated with selective processing of Argonaute (AGO) proteins, and the base preference at miRNA positions 10 and 11 may be related to the specific binding of miRNAs to target genes.

### 2.3. Differentially Expressed miRNAs (DEmiRNAs) between PM_XP and PM_X

**Figure 4:**
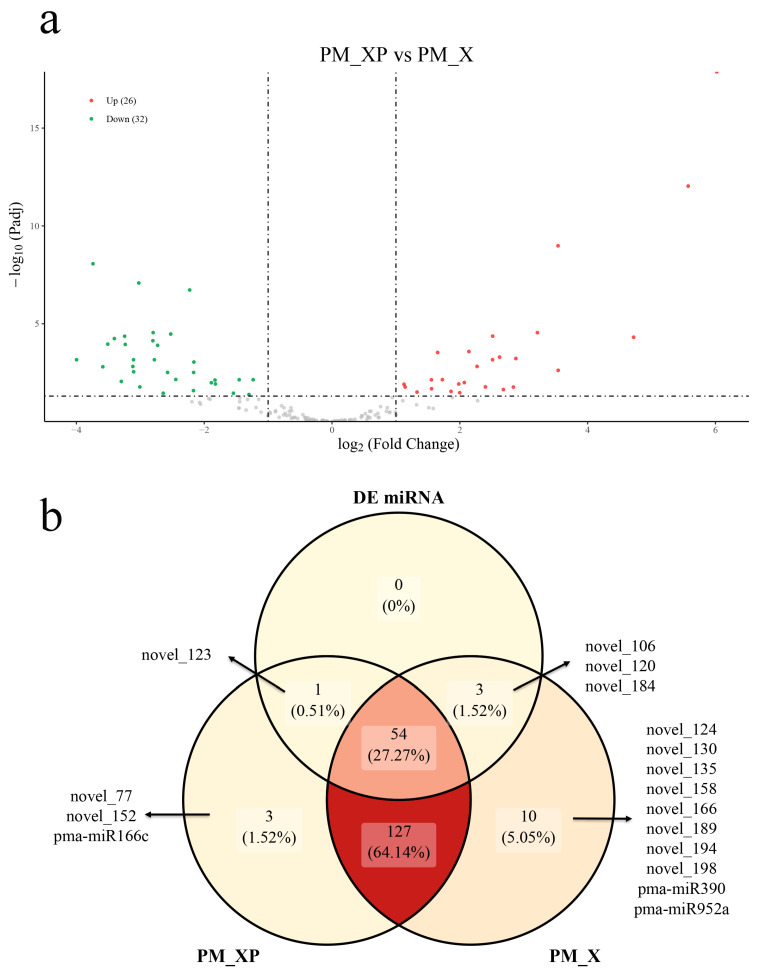
The differentially expressed miRNAs in developing xylem (PM_X) and protoplasts isolated from developing xylem (PM_XP). (**a**) Volcanic diagram of differentially expressed miRNAs. (**b**) Venn diagram of differentially expressed miRNAs.

We found substantial expression differences between PM_XP and PM_X for 58 miRNAs, with 26 miRNAs (23 novel miRNAs and 3 conserved miRNAs) significantly up-regulated in PM_XP compared with PM_X and 32 miRNAs (29 novel miRNAs and 3 conserved miRNAs) significantly down-regulated in PM_XP (Figure 4a. Table S3). We noticed that 4 miRNAs were induced in the cell wall after removal (Figure 4b). DEmiRNAs accounted for 29.29% (58 out of 198) of all miRNAs, and most DEmiRNAs were up-regulated (Figure S3).

### 2.4. Gobal Analysis of miRNA Target Genes via Degradome Sequencing

PsRNATarget was used to predict the target genes of miRNAs [31]. We ultimately obtained 4140 potential target genes of 182 miRNAs (Table S4). CleaveLand4 was used to validate the target genes of the miRNAs [32]. A total of 865 target genes of 133 miRNAs were obtained, including 153 target genes of 20 conserved miRNAs and 712 target genes of 113 novel miRNAs. The complete list of miRNA-mRNA regulatory pairs is shown in Table S5 and Additional file S2. Table 2 lists several target genes of miRNAs.

Analyses of the miRNA target genes found that most of the genes encoded TFs, including *ARFs* and *MYBs* (Table S5). Previous studies revealed complex regulatory relationships between miRNAs and TFs. For example, TFs bind to the upstream promoter region of *MIR* genes and affect the transcription of miRNAs, and miRNAs also act on the mRNAs of TFs and affect their translation process [33]. We found that one miRNA can have many different target genes. For example, novel_42 can target 30 different transcripts, and pma-miR482a can target 32 transcripts. More than one miRNA targeted the same transcript, for example, 3 miRNAs simultaneously targeted Cluster-20177.1 (unknown) (Table S5).

### 2.5. Enrichment Statistics of miRNA Target Genes

We found that “response to hormone”, “small-submit processing”, “rRNA processing”, and “double-stranded DNA binding” were the top 4 terms, and all of these terms had significant *p*-values (Figure S4a). A total of 521 target mRNAs of miRNAs supported by the degradome were assigned to 18 KEGG metabolic pathways. The 3 most abundant pathways were “Folding, sorting and degradation” (50), “Translation” (60), and “Environmental adaptation” (98). These results imply that miRNA target genes play an important role in plant growth and development (Figure S4b).

## 3. Discussion

**Figure 5:**
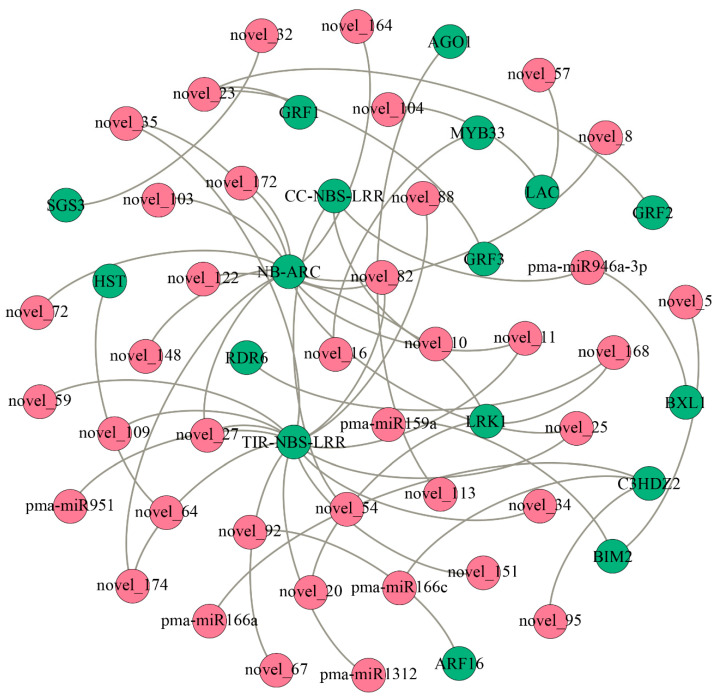
MiRNA-mRNA regulatory modules in developing xylem. The red dots indicate miRNAs, the green dots express target genes, and the connecting line in the middle indicates miRNA-target gene pairs.

Our study systematically identified miRNAs present in the developing xylem of *P. massoniana* and combined sRNA and degradome sequencing techniques to reveal the potential miRNA-mRNA regulatory networks involved in the developing xylem of *P. massoniana*. We identified a total of 198 miRNAs in the developing xylem of *P. massoniana*, including 25 conserved and 173 novel miRNAs. Differential expression analysis found that the expression of 4 miRNAs were induced after cell wall removal, and 58 miRNAs were significantly differentially expressed. An increasing number of studies used single-cell sequencing to study the mechanism of wood formation [34], and our study found that the expression of a small number of miRNAs was induced after the removal of the cell wall, which suggests that we need more thinking in the use of protoplasts for plant signal transduction and cell osmolarity-related studies. A total of 133 miRNAs regulated 841 potential target genes that formed 865 miRNA-mRNA regulatory relationship pairs, and the potential regulatory functions of miRNAs were revealed in this study (Figure 5, Table S5). Two types of target genes were of interest to us, as described below.

### 3.1. Mirna-Target Regulatory Pathways Related to the Stress Response in the Developing Xylem of Masson Pine

Sixteen novel miRNAs and 3 conserved miRNAs showed the ability to target *NBS-LRR* genes. *NBS-LRR* genes give plants the ability to resist various pathogens. The structural features of *NBS-LRR* genes are very conserved in general [35]. We found that 27 *NBS-LRR* genes were regulated by miRNAs in the developing xylem of *P. massoniana*. The products of 20 of these genes contained TIR domains (TIR-NBS-LRR), and 7 did not contain TIR domains (CC-NBS-LRR). Previous studies showed that miRNA were regulatory factors that played a role in regulating the expression of *NBS-LRR* genes in response to stress [36,37,38,39,40]. We found that most of the *P. massoniana NBS-LRR* genes could be regulated by miRNAs, which suggests that the response of cells to stress is relatively strong after the cell wall is removed.

Leucine-rich-repeat receptor-like kinases (LRKs) are the most abundant class of receptor protein kinases. LRKs are widely found in monocots and eudicots and are closely associated with plant growth and development, disease defense and the response to adverse stress conditions. A total of 379, 216, and 300 *LRK* genes were identified in *Populus trichocarpa* [41], *A. thaliana* [42], and *Oryza sativa* [43], respectively. The present study identified 20 *LRK* genes that could be targeted by 3 miRNAs (novel_20, novel_122, and novel_168) in *P. massoniana*, which suggests that miRNAs are involved in plant development via regulation of *LRK* expression.

### 3.2. Conserved Mirna-Mrna Modules between Softwood and Hardwood Formation

Non-coding RNAs are widely involved in various processes of plant growth and development. SRNAs, represented by miRNAs, regulate gene expression at the post-transcriptional level, and a large number of studies also found that miRNAs were involved in the process of wood formation. For example, miRNAs can target *MYB*, *ARF*, and *LAC* genes to regulate the wood formation process.

MYB TFs are widely distributed in higher plants and constitute one of the largest TF families in plants. MYB TFs are involved in most aspects of plant development and metabolism. The present study showed that one of the target genes of novel_16 encoded a MYB TF. As components of the classic regulatory network underlying wood formation, members of the MYB family constitute the second and third levels of the regulatory networks during wood formation. The NAC TFs PtrWND2B and PtrWND6B in *P. trichocarpa* regulate the expression of many wood formation-related TFs, such as PtrMYB3, PtrMYB18, PtrMYB28, PtrMYB75, PtrMYB90, and PtrMYB152, and activate the expression of lignin- and cellulose-related genes [44]. The CCCH zinc finger protein-encoding genes *PdC3H17* and *PdCH18* in *Populus deltoides* are direct targets of PdMYB3 and PdMYB21, which increase the thickness of the xylem secondary walls in stems and promote wood biomass by regulating the expression of downstream secondary wall development-related genes [45]. Similarly, PtoMYB156 in *Populus tomentosa* negatively regulates the expression of *CESA17*, *C4H2* and *GT43B*, and knockdown of the *PtoMYB156* gene leads to an increase in lignin and cellulose contents [46]. Our results showed that novel_16 may be involved in wood formation via the targeting of *MYB* genes.

ARFs are key TFs that regulate growth hormone-related signaling, and their functions are involved in plant growth and development [47]. The *ARF* genes in woody plants species are closely related to the formation of the phloem and xylem, and two genes in *P. trichocarpa* are homologous to *AtARF5*, both of which are related to vascular tissue development and may play a similar role in the development of secondary xylem in *P. trichocarpa* [48]. MiRNAs also regulate *ARF* genes. For example, miR160 targeted *ARF10/16/17* in *A. thaliana* [49]. In this study, we found that *ARF16* is targeted by novel_92. The expression of novel_92 in PM_XP and PM_X was 1030.1195 and 90.919, respectively, and the expression of target genes in PM_XP and PM_X was 1.38 and 9.06 (Cluster-18410.48111), 0.53, and 4.02 (Cluster-18410.55513), and 5.86 and 18.85 (Cluster-18410.73418), respectively. Taken together, these results suggested the expression of novel_92 was induced after the cell wall removal via an unknown mechanism, which increased novel_92 expression and further suppressed the expression of its target gene *ARF*.

**Figure 6:**
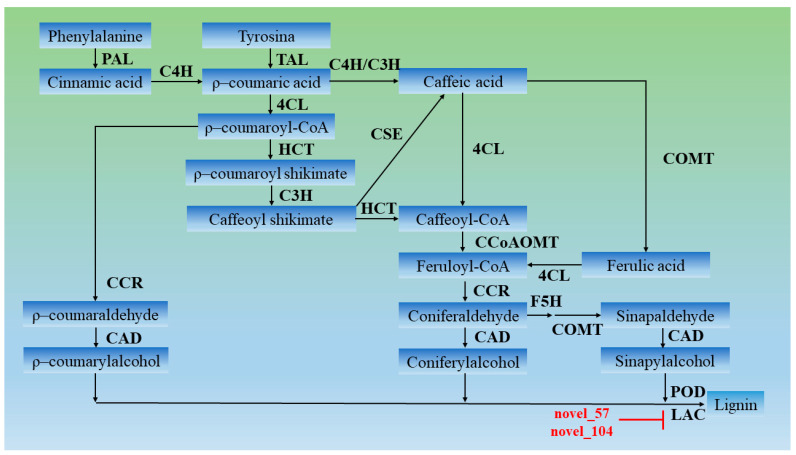
miRNA targets involved in lignin biosynthesis. miRNA cleavage is represented by solid lines with “T” arrows.

**Figure 7:**
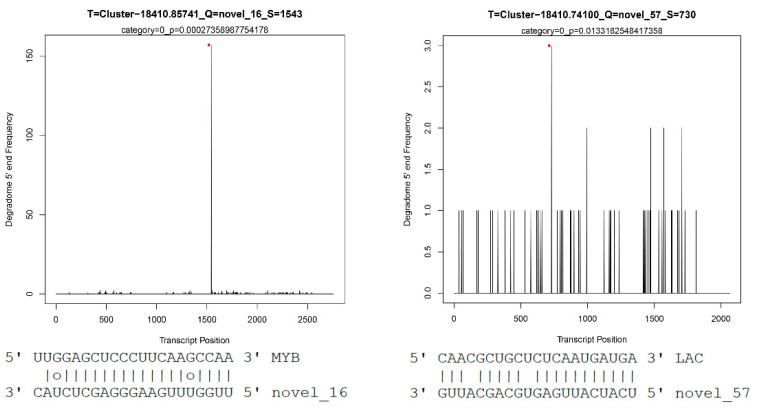
Target plots of novel_16 (**left**) and novel 57 (**right**). The target genes of novel_16 and novel_57 are Cluster-18410.85741 and Cluster-18410.74100, respectively. The horizontal coordinate corresponding to the red dot indicates that the miRNAs cleave the target genes at that position.

**Figure 8:**
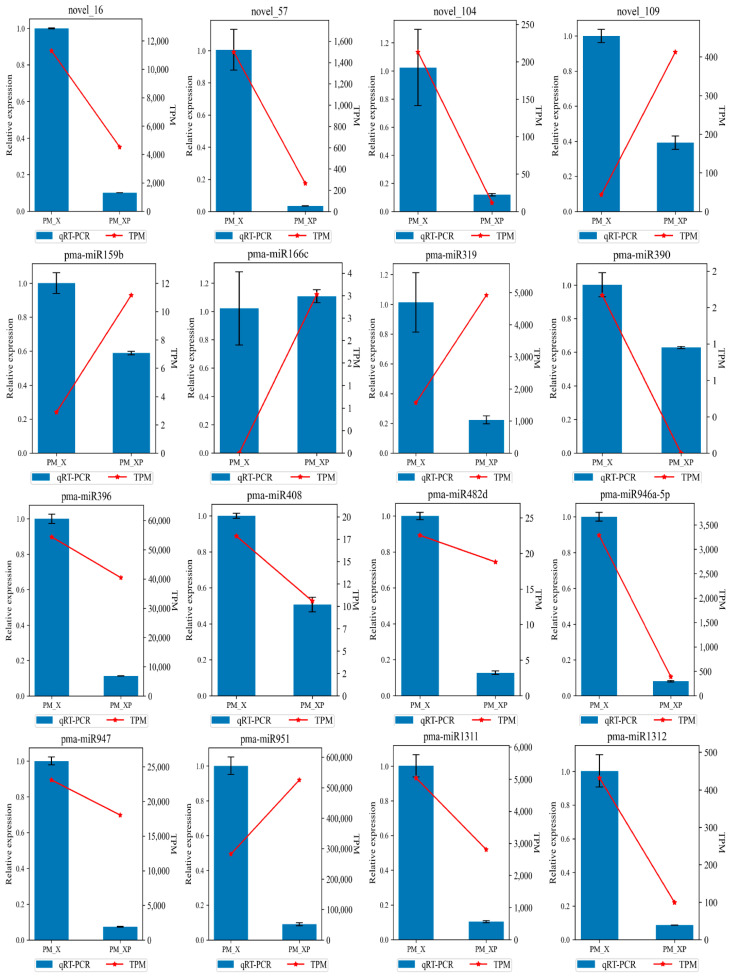
qRT-PCR validation of miRNAs in *P. massoniana*. The blue bars represent the level of expression of miRNAs calculated by qRT-PCR (left *y*-axis). Error bars represent the standard errors of three technical replicates. The relative expression obtained by sRNA sequencing is shown by red lines (right *y*-axis).

Lignin is an important component of SCWs in vascular plants and has important biological functions. Lignin synthesis is a complex and orderly process. Lignin in higher plants is synthesized via the phenylpropanoide pathway and the lignin-specific pathway, and phenylalanine is synthesized via a series of reactions to produce 3 major lignin monomers, which are ultimately polymerized into polymeric lignin under the catalysis of peroxidase (POX) and laccase (LAC) [9]. Numerous studies showed that various non-coding RNAs, such as miR156, miR160, miR164, miR319, and miR397, target *LAC* [50,51,52]. The present study found that novel_57 and novel_104 directly targeted *LAC* to prevent individual lignin monomers from entering the lignin synthesis pathway (Figure 6). Degradome analysis showed that novel_16 and novel_57 and their target genes were strongly associated with category 0 and had significant *p*-values (Figure 7). Normalized expression levels and qRT-PCR showed that the expression of novel_57 and novel_104 in PM_XP was lower than PM_X (Figure 8). The role of lignin in plants is very important, but lignin in the plant cell wall is the main obstacle in the process of making pulp and plant-based biofuels [53]. A high level of lignin in fruits also affects taste, which results in great economic loss. Researchers have used traditional breeding or transgenic breeding techniques to select plants with a low lignin content for the past few decades but found that these practices have several adverse effects on plant development, such as lodging, stunted growth, reduced survival, innate immunodeficiency and reduced yield and biomass [54,55]. Indirect control of lignin biosynthesis via manipulation of miRNAs is a relatively novel approach.

## 4. Materials and Methods

### 4.1. Plant Materials and Sequencing

The developing xylem and its protoplasts were collected from two different genotypes of *P. massoniana* growing on the campus of Nanjing Forestry University. The detailed method for the preparation of the protoplasts of *P. massoniana* is described in a master’s thesis [56]. Briefly, peeled branches with exposed developing xylem were cut into 5–10 cm sections, and these sections were immediately transferred to a freshly prepared enzyme solution and incubated in the dark at 28 °C for 4 h. After the addition of W5 solution to stop digestion, protoplasts were released by filtration and collected by centrifugation.

Total RNA was extracted from developing xylem and its protoplasts of *P. massoniana* using an RNAprep Pure kit (Tiangen, Beijing, China), in accordance with the manufacturer’s instructions. The methods of RNA integrity and concentration assays were reported in our previous publications [57]. Four sRNA libraries were constructed using the NEBNext^®^ Multiplex Small RNA Library Prep Set for Illumina^®^ kit (NEB, Ipswich, MA, USA), and the principle and procedure were described in our previous publications [57]; the prepared sRNA libraries were sequenced using the Illumina HiSeq 2500 platform for 1 × 50 bp sequencing (Novogene, Beijing, China). RNA isolated from the developing xylem and protoplasts isolated from the developing xylem tissue were mixed together equally to construct a degradome library according to previous methods [57]. The prepared libraries were sequenced on the Illumina HiSeq 2500 platform for 1 × 50 bp sequencing (Lianchuan Bio, Hangzhou, China).

### 4.2. Identification of Conserved and Novel Mirnas

The adaptor sequences for sRNA sequencing were obtained using dnapi.py [58], followed by quality control using cutadapt with the detailed parameters -j 0 -a adaptor --quality-base 33 -m 18 -M 30 -O 4 --discard-untrimmed -q 20 --max-n 0 --quiet -o Sample_name.Clean.fq Sample_name.Raw.fq.gz [59]. The detailed methods used to identify miRNAs were described in our previous research [57]. Briefly, we filtered reads of low quality and reads derived from other RNAs then used miREvo and miRDeep2 software to predict *P. massoniana* miRNAs [60,61].

### 4.3. Identification of DEmiRNAs

To identify DEmiRNAs, we normalized the expression levels of miRNAs in PM_X1, PM_X2, PM_XP1, and PM_XP2 samples via TPM values. We then used DEseq2 software to perform a differential expression analysis of miRNAs [62]. MiRNAs with |log2 (fold change)| value > 1 and an adjusted *p*-value < 0.05 were identified as differentially expressed. Cluster analysis was subsequently performed by TBtools [63].

### 4.4. Identification of miRNA Target Genes

First, we used the online software psRNATarget to predict the target genes of miRNAs [31]. The prediction criterion was a seed region of the 2nd~8th nt from the end of the miRNA 5′ end, and no mismatch was allowed in the seed region. The raw data of degradome sequencing were quality controlled using fastp [64]. We used CleavelLand4 with parameters -t -c 2 to analyze the degradome sequencing data and comprehensively predict the target genes of miRNAs [32]. Transcriptome data were downloaded from the SRA database with the accession numbers SRR12596930-SRR12596933, and the data processing methods are described in detail in Xu’s master’s thesis [56].

### 4.5. Enrichment Analysis of miRNA Target Genes

We used Blast2GO to annotate the target genes of miRNAs [65], a custom-made Python script was then used to filter and sort the annotation results, and the enrichment analysis was performed via clusterProfiler [66].

### 4.6. qRT-PCR Validation

The reference genes for qRT-PCR performed in this study were *UBI4* and *TUA*, and the sequences of the primers used are shown in Table S6. According to the Mir-X miRNA First-Strand Synthesis kit instructions (TAKARA, Dalian, China), 1 μg of total RNA (containing miRNA) was first reverse transcribed into cDNA and verified by qRT-PCR using the specific mRQ 3′ primers in the kit with the corresponding miRNA sequences. Reactions were performed on ViiA™ 7 real-time PCR Systems (Applied Biosystems, Waltham, MA, USA), and three technical replicates were performed for each sample, and relative expression was calculated using the 2^−ΔΔCt^ method.

## 5. Conclusions

We identified miRNAs in the developing xylem of *P. massoniana* and validated miRNAs by qRT-PCR. We found that 4 miRNAs (novel_16, novel_57, novel_92, and novel_104) may be involved in *P. massoniana* wood formation via the targeting of *MYB*, *ARF*, and *LAC*. Our results revealed a potential regulatory network in which miRNAs were involved in the developing xylem of *P. massoniana*, revealed possible conserved miRNA-mRNA modules involved in softwood and hardwood formation, and provided new insights into wood formation in coniferous species.

## Figures and Tables

**Table 1:** Summary of sRNA sequencing data from four RNA libraries in the developing xylem (PM_X) and protoplasts isolated from developing xylem (PM_XP).

Category	PM1_X	PM2_X	PM1_XP	PM2_XP	Average
Raw Reads	19,122,111	21,352,761	17,656,738	17,053,919	-
Q20	97.67%	97.70%	97.67%	97.67%	97.68%
Q30	94.80%	94.83%	94.72%	94.74%	94.77%
Clean Reads	18,456,504	20,654,598	17,068,608	16,485,018	-
Clean Reads of sRNA	14,681,663	17,072,786	9,997,932	10,846,339	-
Mapped sRNA Reads	11,619,254	12,936,717	7,439,780	7,686,301	-
Mapped Rate	79.14%	75.77%	74.41%	70.87%	
Conserved miRNA Reads	341,974	108,456	93,012	76,618	-
Uniq Conserved miRNA Reads	464	316	332	307	-
Conserved miRNA Hairpin	28	24	26	26	-
Conserved miRNA Mature	24	20	22	21	-
Novel miRNA Reads	371,500	182,024	52,850	50,364	-
Uniq Novel miRNA Reads	2454	1481	1556	1442	-
Novel miRNA Mature	165	162	152	153	-
Novel miRNA Hairpin	173	171	163	161	-

**Table 2:** Target genes of conserved and novel miRNAs identified in developing xylem of *P. massoniana* by degradome sequencing.

miRNA	Target Transcript	Transcript Annotation	Degradome Category	DegradomePval
pma-miR951	Cluster-18410.91163	TIR-NBS-LRR	0	0.022455112
pma-miR946a-3p	Cluster-17513.1	CC-NBS-LRR	0	0.006001718
pma-miR946a-3p	Cluster-18410.68882	CC-NBS-LRR	0	0.053772939
pma-miR946a-3p	Cluster-6417.0	CC-NBS-LRR	0	0.003278143
pma-miR946a-3p	Cluster-6417.1	CC-NBS-LRR	0	0.003550836
pma-miR946a-3p	Cluster-6417.2	CC-NBS-LRR	0	0.006545539
pma-miR946a-3p	Cluster-18410.69983	BXL1	0	0.025393001
pma-miR482a	Cluster-18410.4683	Zinc Ion-binding protein	0	0.009260186
pma-miR396	Cluster-18410.26942	CYCT1-3	2	0.045402037
pma-miR166c	Cluster-18410.59035	C3HDZ2	0	0.00027359
pma-miR166a	Cluster-18410.60044	C3HDZ2	2	0.011549044
pma-miR166a	Cluster-18410.65256	C3HDZ2	2	0.022964707
pma-miR159a	Cluster-18410.64102	BIM3	1	0.000935432
pma-miR1314	Cluster-22970.0	BAM3	0	0.00027359
pma-miR1312	Cluster-18410.35178	TIR-NBS-LRR	1	0.018543312
novel_95	Cluster-18410.58110	C3HDZ2	0	0.000820545
novel_92	Cluster-18410.48111	ARF16	0	0.00109391
novel_92	Cluster-18410.55513	ARF16	0	0.002186624
novel_92	Cluster-18410.62143	ARF16	0	0.000547105
novel_92	Cluster-18410.71517	ARF16	0	0.001367201
novel_92	Cluster-18410.73418	ARF16	0	0.005185428
novel_92	Cluster-18410.73420	ARF16	0	0.003823454
novel_92	Cluster-18410.73422	ARF16	0	0.004913182
novel_82	Cluster-18410.37093	TIR-NBS-LRR	1	0.022210506
novel_82	Cluster-18410.114635	TIR-NBS-LRR	1	0.031318655
novel_82	Cluster-18410.19951	TIR-NBS-LRR	0	0.011156025
novel_67	Cluster-18410.75829	TIR-NBS-LRR	2	0.034248531
novel_59	Cluster-18410.68783	TIR-NBS-LRR	2	0.034248531
novel_59	Cluster-18410.68790	TIR-NBS-LRR	0	0.000547105
novel_57	Cluster-18410.74100	LAC	0	0.013318255
novel_54	Cluster-18410.61870	CC-NBS-LRR	0	0.027257968
novel_5	Cluster-18410.33788	BIM2	2	0.05642673
novel_35	Cluster-18410.30641	TIR-NBS-LRR	0	0.097521346
novel_34	Cluster-18410.90022	TIR-NBS-LRR	0	0.017359701
novel_32	Cluster-18410.57154	SGS3	0	0.076790575
novel_27	Cluster-18410.37995	TIR-NBS-LRR	2	0.078095614
novel_25	Cluster-18410.82637	TIR-NBS-LRR	2	0.011549044
novel_20	Cluster-18410.84714	LRK1	0	0.003550836
novel_174	Cluster-18410.33579	TIR-NBS-LRR	0	0.035210347
novel_174	Cluster-18410.101866	TIR-NBS-LRR	0	0.009531243
novel_168	Cluster-18410.19527	RDR6	0	0.082080284
novel_168	Cluster-18410.92321	LRK1	0	0.020044798
novel_16	Cluster-18410.85741	MYB33	0	0.00027359
novel_16	Cluster-18410.85743	MYB33	0	0.000547105
novel_16	Cluster-18410.27112	MYB33	0	0.00109391
novel_151	Cluster-18410.19951	TIR-NBS-LRR	2	0.078095614
novel_146	Cluster-18410.109049	TIR-NBS-LRR	0	0.002186624
novel_122	Cluster-18410.49874	LRK1	1	0.010241759
novel_113	Cluster-18410.63994	AGO1	0	0.004913182
novel_113	Cluster-18410.63996	AGO1	0	0.004640862
novel_11	Cluster-18410.33711	TIR-NBS-LRR	2	0.0673241
novel_11	Cluster-18410.33065	TIR-NBS-LRR	0	0.019776619
novel_109	Cluster-18410.27403	TIR-NBS-LRR	2	0.034248531
novel_10	Cluster-18410.113971	CC-NBS-LRR	0	0.000547105

BXL1 (Beta-D-xylosidase 1), CYCT1-3 (Cyclin-T1-3), C3HDZ2 (Class III homeodomain-leucine zipper protein), BIM3 (BES1-interacting Myc-like protein 3), BAM3 (Leucine-rich repeat receptor-like serine/threonine-protein kinase), ARF16 (auxin response factor 16), LAC (laccase), SGS3 (protein suppressor of gene silencing 3), LRK1 (L-type lectin-domain containing receptor kinase), AGO1 (Argonaute 1).

## Data Availability

All the data are shown in the main manuscript and in the Supplementary Materials. The sRNA sequencing described in this manuscript were submitted to the National Center for Biotechnology Information (NCBI) under accession codes SRR15384540-SRR15384543.

## Supplementary Materials

The following are available online at https://www.mdpi.com/article/10.3390/ijms221810154/s1, Figure S1: Length distribution of sRNA reads in PM_XP and PM_X; Figure S2: Length distribution of conserved miRNAs (I) and novel miRNAs (II); Figure S3: Expression patterns of differentially expressed miRNAs in PM_XP and PM_X; Figure S4: Annotation information for miRNA target genes in *P. massoniana*; Table S1: Detailed information of conserved and novel miRNAs; Table S2: Detailed information of conserved and novel miRNA expression; Table S3: Detailed information of differentially expressed miRNAs; Table S4: Target genes of miRNAs identified in *P. massoniana* by psRNATarget; Table S5: Target genes of miRNAs identified in *P. massoniana* by degradome sequencing; Table S6: Primers used in this study; Additional file 1: Secondary structures of miRNA precursors; Additional file 2: Target plots of *P. massoniana* degradome sequencing.
