# Supplementary figures and images for: Uncovering miRNA-mRNA Regulatory Modules in Developing Xylem of Pinus massoniana via Small RNA and Degradome Sequencing
Source: Int J Mol Sci. 2021 Sep 21;22(18):10154. doi: 10.3390/ijms221810154 (PMC8472836; doi:10.3390/ijms221810154)

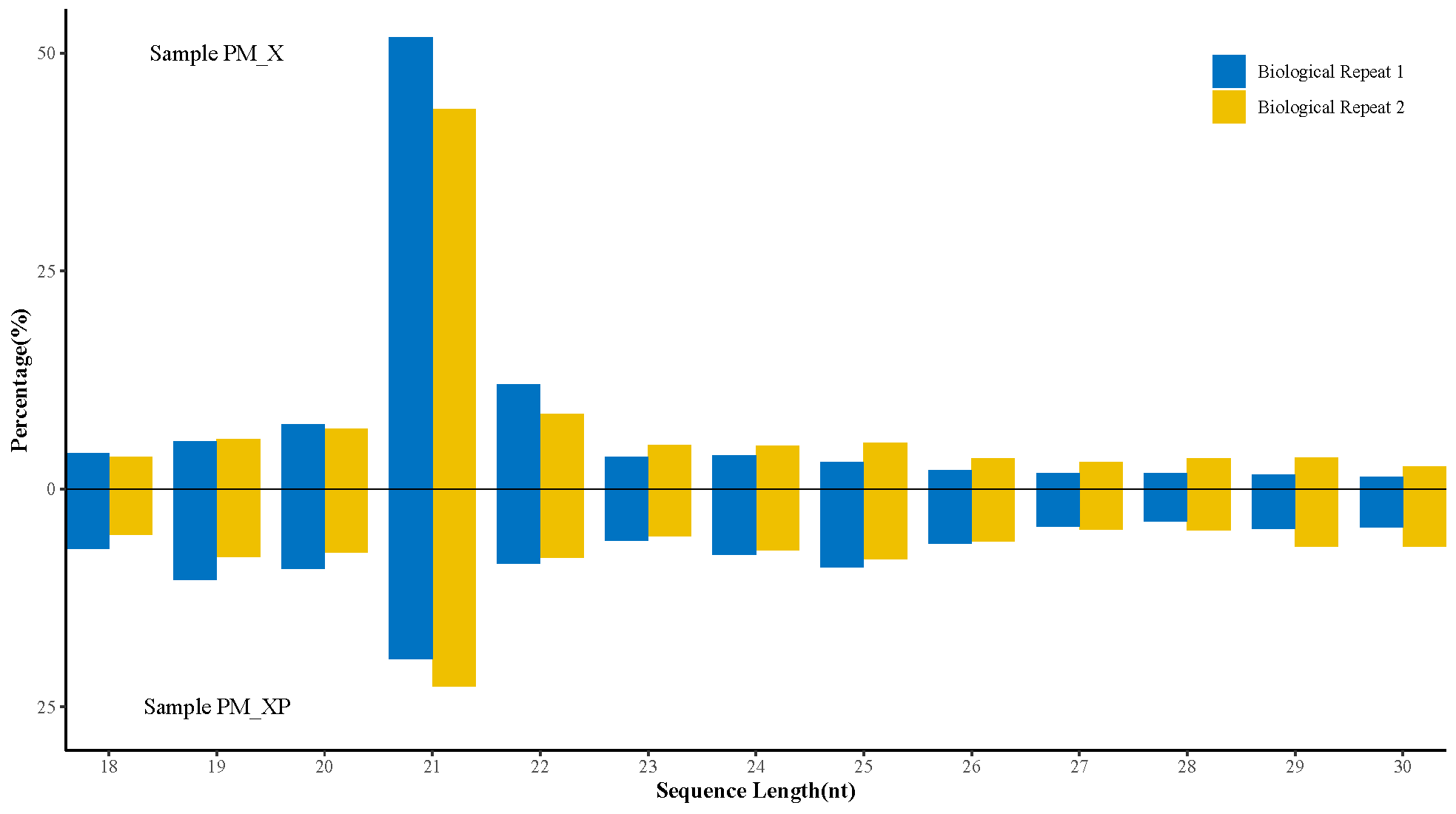

Supplement: Supplementary file 1 [file ijms-22-10154-s001.zip › ijms-1365557-supplementary/Figure S1.tiff]

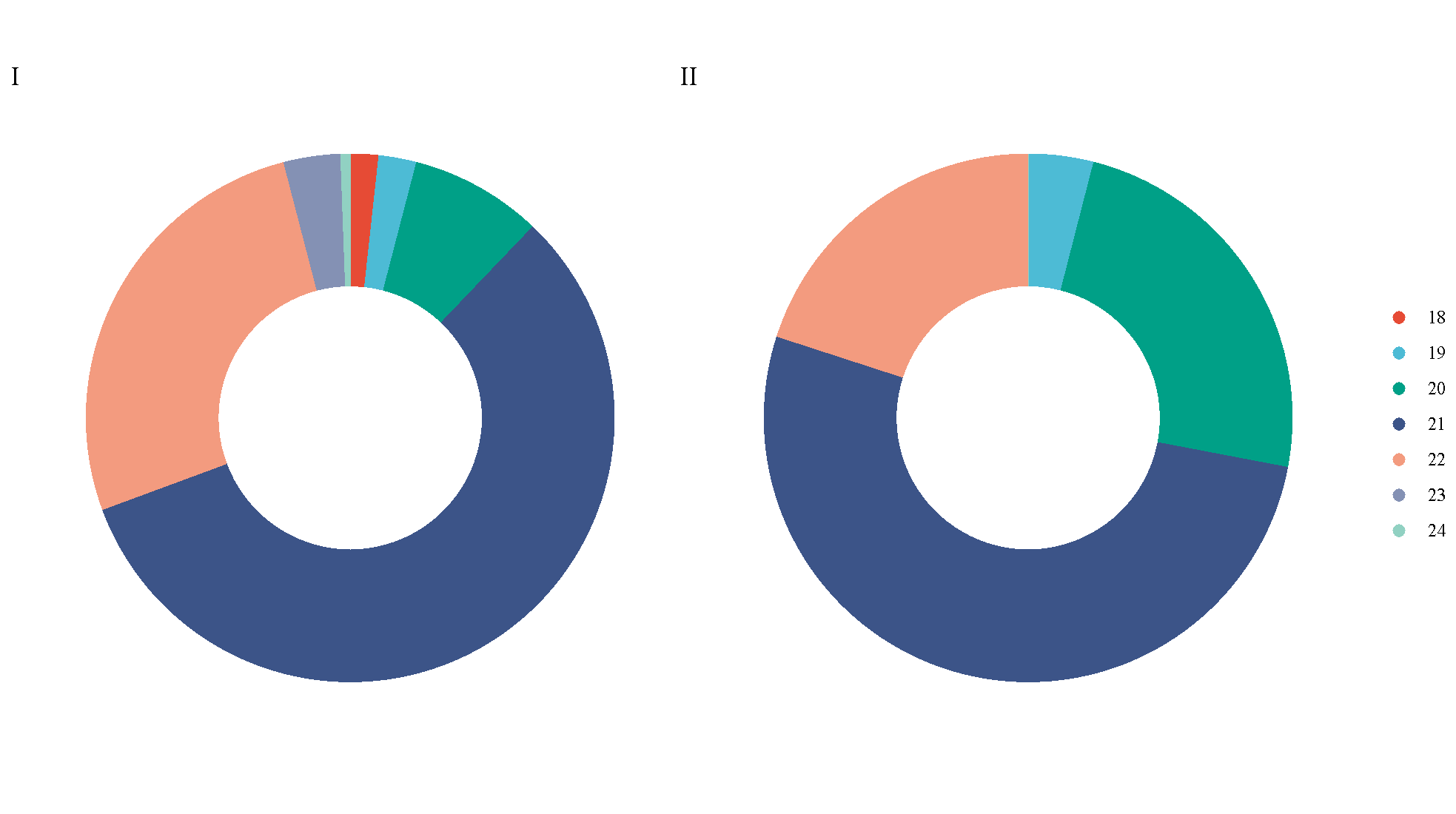

Supplement: Supplementary file 1 [file ijms-22-10154-s001.zip › ijms-1365557-supplementary/Figure S2.tiff]

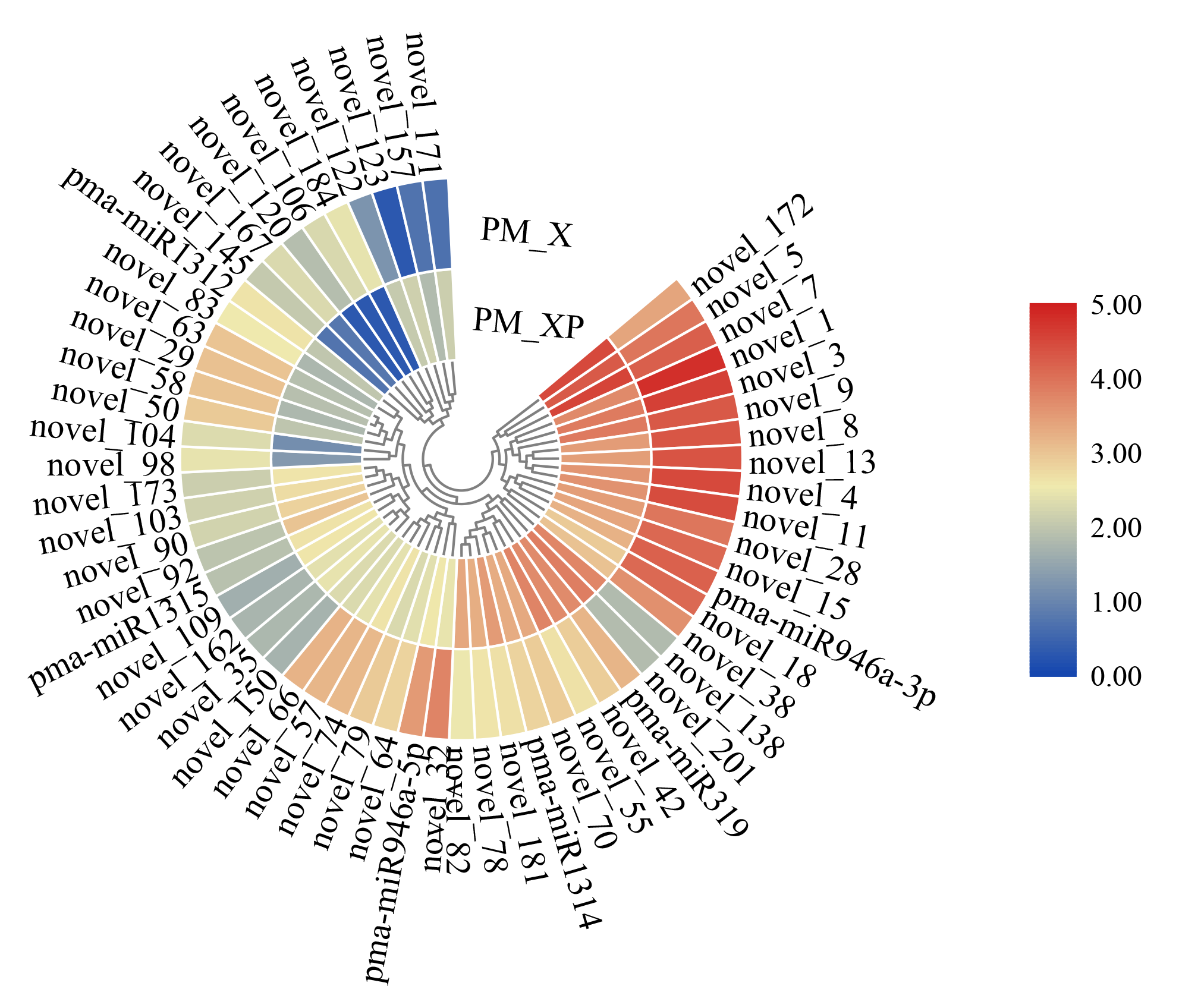

Supplement: Supplementary file 1 [file ijms-22-10154-s001.zip › ijms-1365557-supplementary/Figure S3.tiff]

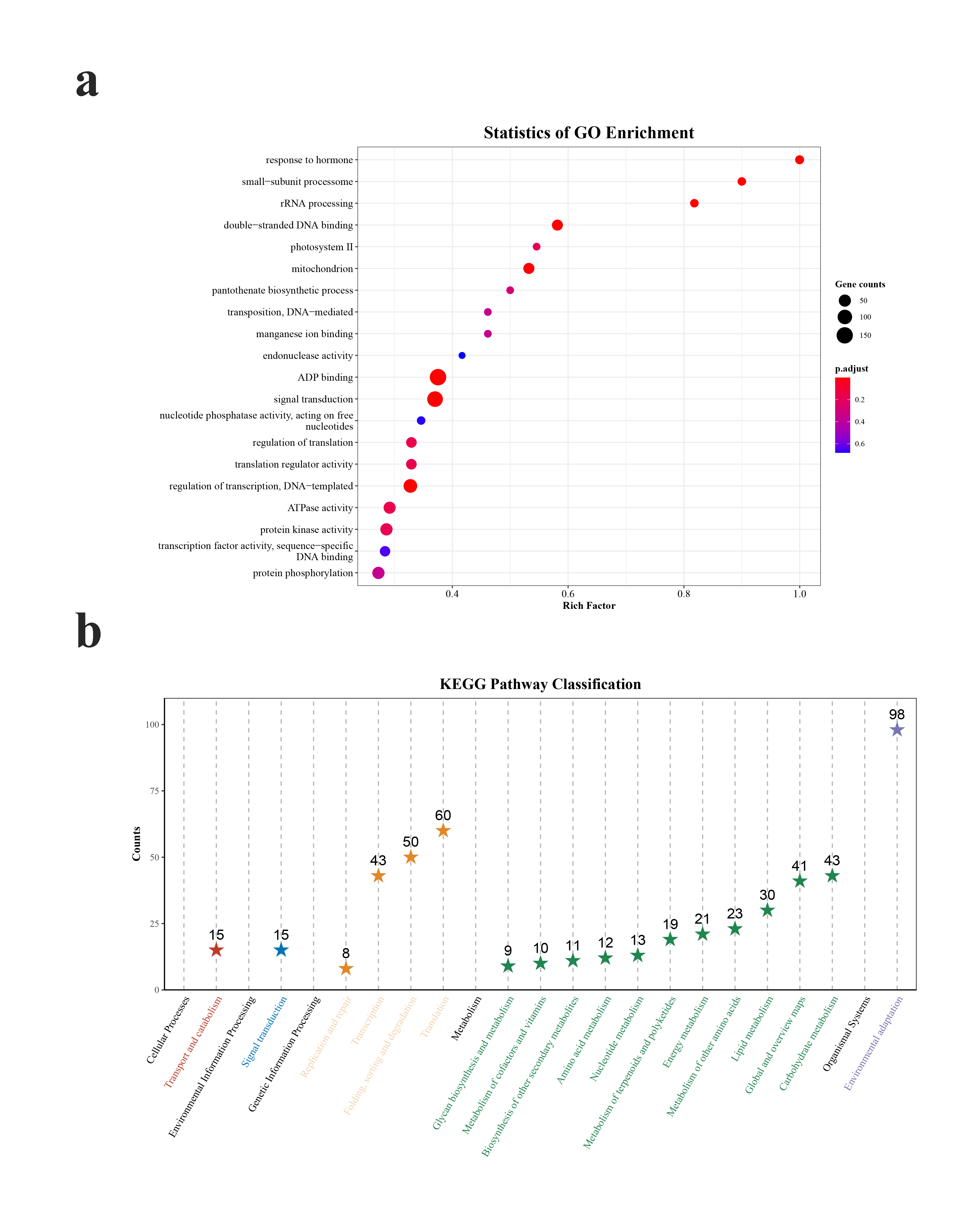

Supplement: Supplementary file 1 [file ijms-22-10154-s001.zip › ijms-1365557-supplementary/Figure S4.tiff]
